# Is there a relation between carpal tunnel syndrome and trigger finger?

**DOI:** 10.1186/1753-6561-9-S3-A65

**Published:** 2015-05-19

**Authors:** Shaher El-Hadidi

**Affiliations:** 1 Jordan University Hospital, Amman, 11942, Jordan

## Introduction

Idiopathic carpal tunnel syndrome is the most common upper limb entrapment neuropathy.

The prevalence in the general population is 2.7%. Trigger digit is also a common clinical disorder, which is characterized by painful locking of the involved digit. Both disorders are important causes of occupational absenteeism and disability, and often coexist in the same hand.

Trigger finger and carpal tunnel syndrome together are in all probability the most common conditions treated by a hand surgeon. Previous studies have suggested a significant concurrence rate between these conditions, but the implications of this have not been fully explored.

Some studies hypothesize that surgery for one entity will cause the other or that the carpal tunnel syndrome is associated with the appearance of trigger digits as a separate entity.

The etiology of both TF and CTS is unknown. It is possible that there is a common etiology present in many cases, and the relationship to diabetes may help shed light on this.

The goal of this study is to quantify the rate of trigger fingers after open carpal tunnel release.

## Materials and methods

A sampling of 565 consecutive cases was obtained from January 2002 to June 2011 using a retrospective and observational study. These were the patients who were operated upon for carpal tunnel syndrome by open release of transverse carpal ligament. All the surgeries were performed under local anesthesia infiltration in most cases and under general anesthesia in some cases at the Jordan University Hospital.

The diagnosis was made on the basis of a standard history and physical exam performed by the authors.

CTS was defined by the presence of at least two of the following: numbness or tingling in the median nerve distribution, diminished median nerve motor function, or a positive Tinel's or Phalen's test. All CTS patients had nerve conduction tests (NCTs) and electromyography (EMG) consistent with CTS.

Cases of TF were defined by a history and/or presence of catching, clicking or locking and tenderness over the A1 pulley.

Demographic data was obtained from the patients.

## Results

We obtained information from 565 patients who had carpal tunnel syndrome that was treated surgically by open release. 103 patients were males (18.23%); of those; the affected side was right 37 (36%), left 20 (19%) and, bilateral 46 (45%). 462 patients were females (81.77%); of those; the affected side was right 108 (23%), left 64 (14%), and bilateral 290 (63%).

The Average age for male patients was (55.8 years), with range of (22-89 years).The average age for female patients was (53.4 years), with range of (18 – 91 years).

113 patients were diabetic (20%): of those; 28 (18%) were males, and 85 (82%) were females.

**Table 1:** Trigger fingers after open CTS release and their distribution

Triggering post CTS release:59	Male:9	Female:50
1-Thumb:15	0	15
2-Index:23	5	18
3-Middle:17	2	15
4-Ring:3	1	2
5-Little:1	1	0

Triggering at presentation occurred in 73 patients (13%); 19 were males and 54 were females.

The average duration of symptoms pre-op was 10.77 months, with range of 1 month.-72 months. The average duration between releases in cases of bilateral CTS was 1.61 months., with range of 1-6 months.

Triggering after open carpal tunnel release occurred in 59 patients (10.44%); 9 were males, and 50 were females.

Diabetes Mellitus was observed in 11 patients in whom triggering occurred after open carpal tunnel release (18.6%).

## Discussion

The etiology of CTS is multi-factorial. Patients with idiopathic carpal tunnel syndrome are predisposed to the development of trigger digit. The incidence in various reports ranges from 0.2% to 22%. However, in most of these studies, trigger digit was only evaluated at the time of the first visit to the hospital for carpal tunnel syndrome.

Trigger digit occurring after carpal tunnel release was examined in the retrospective study by Hombal et al. He reported an incidence of 22% within one year of carpal tunnel release. Hayashi et al suggest that carpal tunnel release surgery is a risk factor for the onset of trigger digit.

Surgery may change the environment inside and near the carpal tunnel. Biomechanically, the flexor tendons at the wrist are known to displace anteriorly after division of the transverse carpal ligament. When this ‘‘bowstringing” occurs, the angle of attack of the flexor tendon against the A1 pulley increases, which may cause a greater frictional and compressive force between the flexor tendons and the A1 pulley.

This increased force at the tendon-pulley interface may lead to trigger digit. Fibrocartilagenoumetaplasia is known to develop at the site of increased compressive forces on connective tissues, and has been reported at the friction surface of the A1 pulley taken from a patient with trigger digit.

Surgery may accelerate the development of trigger digit when carpal tunnel syndrome is mild to moderate. In severe disease, other factors as yet unknown, such as hypertrophy of the flexor tenosynovium, may mask the effect of surgery.

Mackinnon has observed that after CTS surgery a possible trigger finger pathology, that had not been present prior to surgery, can occur.

In our study, we found that trigger finger after open carpal tunnel syndrome occurred in 59 patients (10.44%), diabetes mellitus was observed in 11 patients only (18.6%).

## Conclusion

CTS and TF commonly occur together at presentation though the symptoms of one condition will be more prominent. Trigger finger after open carpal tunnel release is possible to occur. Our results support a common local mechanism that may be unrelated to the presence of diabetes.

